# Integrated care: learning between high-income, and low- and middle-income country health systems

**DOI:** 10.1093/heapol/czx039

**Published:** 2017-11-24

**Authors:** Sandra Mounier-Jack, Susannah H Mayhew, Nicholas Mays

**Affiliations:** 1 Department of Global Health and Development, Health Policy; 2 Department of Health Services Research and Policy, Health Policy and Reproductive Health, London School of Hygiene & Tropical Medicine, London, UK

**Keywords:** Health systems, integration

## Abstract

Over the past decade, discussion of integrated care has become more widespread and prominent in both high- and low-income health care systems (LMICs). The trend reflects the mismatch between an increasing burden of chronic disease and local health care systems which are still largely focused on hospital-based treatment of individual clinical episodes and also the long-standing proliferation of vertical donor-funded disease-specific programmes in LMICs which have disrupted horizontal, or integrated, care. Integration is a challenging concept to define, in part because of its multiple dimensions and varied scope: from integrated clinical care for individual patients to broader systems integration—or linkage—involving a wide range of interconnected services (e.g. social services and health care). In this commentary, we compare integrated care in high- and lower-income countries. Although contexts may differ significantly between these settings, there are many common features of how integration has been understood and common challenges in its implementation. We discuss the different approaches to, scope of, and impacts of, integration including barriers and facilitators to the processes of implementation. With the burden of disease becoming more alike across settings, we consider what gains there could be from comparative learning between these settings which have constituted two separate strands of research until now.

## Introduction

The quest for more integrated care has become a prominent policy theme in high-income country (HIC) health care systems over the past decade. It is considered as a means to provide more patient-focused, coordinated care, and a more efficient health care system, in contexts where people living longer with long-term multiple conditions have emerged as among the main cost drivers. In low- and middle-income countries (LMICs), perennial debates about how vertical health programmes impede coordinated health service delivery have led to divergent views on the processes and benefits of better integration of services (Mills 1983; WHO 1996; Unger et al. 2003). A vertical programme tends to provide the ‘solution of a given health problem by means of single-purpose machinery’ (Gonzalez 1965). By contrast, a horizontal approach favours more holistic ways of dealing with health problems by investing in strengthening existing health services over the long term. Over time, interest in more horizontally integrated care has grown, spurred by the aspiration to provide better continuity of care for patients affected by conditions that are increasingly seen as chronic and often occurring in combination, such as HIV and tuberculosis (TB), or TB and diabetes. An additional more recent push in LMICs has been a global commitment to ensure that countries and external funders alike optimize constrained resources (OECD 2005, 2008).

In all countries, the rationale for more integrated care shares the common goals of improving quality and continuity while reducing costs, often through an enhanced role for primary and community-based care over specialized and hospital-based models. Despite such similar rationales, two very distinct and separate strands of research on integration of care have developed in HICs and LMICs. This paper starts to map the similarities and differences between these bodies of knowledge and the contexts in which they were generated in order to come to a richer understanding of each, and in so doing explore whether lessons for policy and practice can be learned across these diverse settings.

## Integration form, approaches and scope across countries

### High-income countries

Integrated care takes many different forms and remains challenging to define. A review published in 2009 (Armitage et al. 2009) found no fewer than 175 definitions and concepts of integration, although they had many elements in common. One definition potentially useful across many settings is integration as a ‘tailor-made combination of structures, processes and techniques to fit the needs of the people and populations across the continuum of care’ (Valentijn et al. 2013). It focuses on the patient, not only as a beneficiary but also as an integrator of his/her own services. It spells out the need to deliver the ‘right care at the right time’ or, in the vocabulary of the recent English National Voices definition, the capacity to address needs in response to a set of ‘I statements’ setting out what users want from their care (e.g. ‘I work with my team to agree a care and support plan’; ‘My care plan is clearly entered on my record.’) (National Voices 2015). However, this definition may mean that integrated care becomes entirely person- or context-specific, as it responds to patients’ needs through highly tailored services and clinical pathways. This raises challenges for both defining and evaluating integrated care models in any sort of generalizable way.

There are several ways to make more specific the definition of service integration in HICs. The first is to define the population of interest. For instance, integrated care can target a whole population (e.g. integration between health and social care, integration between physical and mental health for all users); a defined population (e.g. frail elderly) across a range of diseases; or a group affected by one or multiple conditions (e.g. people living with HIV; patients with two or more long-term conditions). The second is to assess how strongly elements of service delivery are coordinated, aligned or integrated. This can relate to staff (e.g. whether they work in fully managerially integrated teams vs simply being co-located with staff separately managed), information systems (e.g. shared individual patient records across organizations vs limited forms of aggregated data linkage), governance and policies (e.g. shared treatment protocols and aligned performance management vs common high-level goals), culture and leadership (e.g. shared values vs periodic common training opportunities across organizations) and financial management (e.g. pooling of funds across services and aligned reimbursement incentives such as use of a single capitation budget across different providers vs maintaining separate budgets and payments methods) (Mason 2013).

Among conceptual frameworks that have been used in HICs to describe and evaluate integrated care, the one defined by Valentijn et al. (2013) has proved valuable in distinguishing the different ingredients of integration, listed above, that are needed at different levels in the health care system. It sets out clearly the multi-faceted and complex challenges of bringing about care integration.

### Low- and lower-middle-income countries

The debate between proponents of vertical and horizontal approaches to health care delivery in LMICs can be traced back to 1960s when the merits of interventions focused on specific (vertical) high-priority services (e.g. maternal and child health) vs a more holistic (horizontal, integrated) approaches to primary health care were first contrasted. This culminated in the 1978 Alma-Ata Declaration on comprehensive primary health care which became the defining objective for horizontal, integrated service delivery for a generation (Mills 1983; WHO 1996; Unger et al. 2003). Beset by the challenges of implementing this holistic agenda in resource-constrained settings, however, the proponents of vertical service delivery responded by promoting the concept of Selective Primary Health Care (PHC) as a more feasible and therefore potentially more effective approach to improving care, especially in the shorter term. As experiences were documented, thinking became more nuanced and took on a systems lens, using the language of continuity between levels of care (understood mainly as being through referral pathways) rather than an assumption that the primary health care level alone could provide a comprehensive integrated package of care. Following these debates, WHO (2008) was defining integrated service delivery as ‘*the management and delivery of health services so that clients receive a continuum of preventive and curative services, according to their needs over time and across different levels of the health system*’.

In a systematic review of integration in LMICs from 2006, Briggs and Garner (2006) identified three types of integration (though there is some overlap between them). In the first, the integrator is generally a communicable disease programme often funded by external agencies (e.g. encouraging mothers attending a child immunization clinic to use family planning services at the same time; or conducting TB testing and treatment within HIV/AIDS programmes to address co-infection). The second provides an integrated service to replace previously separate services and facilities (e.g. providing sexually transmitted infection (STI) testing and treatment within family planning services rather than at separate infectious disease clinics; or providing family planning services at a maternal and child health centre rather than at a separate family planning clinic). The third covers the development of packages which integrate services for a specific population (e.g. the Integrated Management of Childhood Illnesses (IMCI) programme that aims to provide enhanced childcare services vs routine (fragmented) child health care).

These models of integration in low-income settings generally focus on health care and seldom attempt to integrate with non-health (e.g. social) services. Most exceptions to this are in the fields of HIV and gender-based violence in which wider socio-structural drivers are more frequently recognized and interventions have included a range of supports to empower women (Abramsky, 2014). Adolescent reproductive health programmes have also recognized the need to partner with Ministries of Education and Youth and Sports to provide more holistic care beyond the health sector to address poor adolescent reproductive health outcomes (Mayhew et al. 2015). Such projects have encompassed clinic-based care alongside school-based debate and teaching on sexual and reproductive health, women’s rights and the role of health services. Some have sought to engage young people, especially boys, in taking greater responsibility for their actions including in relationships and families.

## Scope of integration across income settings

In general, compared with HIC integration approaches, those in LMICs have been narrower in scope and arguably less ambitious. Integration in LMICs has tended to focus on integrating specific clusters of health services for specific populations at the service delivery level—frequently in response to external donor priorities and accountability requirements. The focus in HICs is often on managing multiple morbidities across a wider patient sub-population or includes attempts to coordinate a wider range of services including those outside the health care system. Table 1 compares the target populations, expectations of integration and examples of care between income settings.

**Table 1.** Comparison of typical integrated care programmes in HICs and LMICs

Integration	High-income countries	Low- and middle-income countries
Target population	Elderly, high-cost population People with long-term conditions Complex patients (e.g. combination of physical and mental health conditions, patients needed high intensity of healthcare) Children (vulnerable, at risk) People with mental health needs	Pregnant women and women of reproductive age Children People with infectious diseases (e.g. STI, HIV, TB) Vulnerable or hard to reach populations (e.g. sex workers, drug users)
Expectations of integration	Improve outcomes Improve patient experience Improve quality of care Reduce costs (more efficient use of existing resources) Reduce unplanned admissions Reduce length of stay Reduce residential care Increase community care	Increase access for an increased range of services to a specific population (basic care package) Increase convenience for patients and community (by reducing separate visits) Increase uptake of some services (e.g. family planning) by tagging on to other services (e.g. HIV care) Improve efficiency; share scarce resources between programmes Provide a way to allocate resources for under funded programmes (e.g. adolescent health)
Examples of models of care	Case finding Care planning (including escalation plan) Care co-ordination with regular review Multi-disciplinary teams to deliver care in the community Patient streaming (risk stratification) Virtual wards/hospital at home Patient self-management of long-term conditions	Integrated HIV and reproductive health services Integrated outreach services (eg. vaccinations, Vitamin A, de-worming medicines, bednets) TB and HIV integrated care Child Health Days Integrated Management of Childhood Illness (IMCI) Screening of specific diseases (eg HIV, Syphilis) at antenatal care clinics

Sources: Briggs and Garner (2006), Atun et al. (2008), Partapuri et al. (2012), Curry and Ham (2010), Erens et al. (2016), Ham et al. (2011), Mangiaterra (2014) and Vasan et al. (2014).

**Table 2.** Conceptual framework of integrated care, adapted from Valentijn et al. (2013)

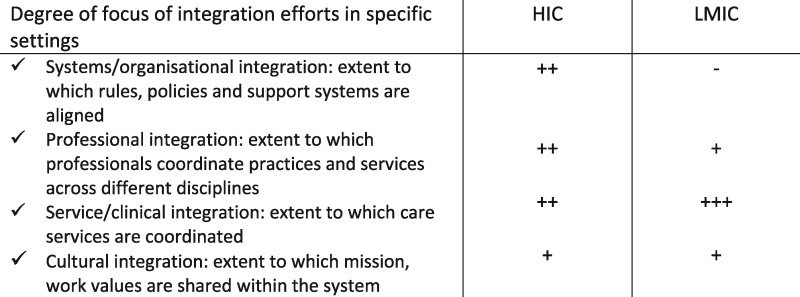

As shown in Table 1, the focus of integrated care varies depending on context. As noted earlier, the target populations are different and the expectations of integration in LMICs are particularly about improving funding and efficiency in order to improve access to, and uptake of, services, while in HICs the emphasis is more on improving quality and patient experience, and reducing dependence on perceived more costly hospital and residential care. This reflects the different starting points of systems at the two different income settings in terms of prior investment, current capacity and population needs. Thus the models of care also have a different emphasis: in LMICs, they are targeted at investing further in, and re-designing priority services (e.g. maternal health, child health and communicable diseases), while in HICs the care models are aimed at changing the way the system works (e.g. case-finding, care planning, case management, multi-disciplinary team working, patient streaming through risk stratification) as opposed to how individual services interact.

Although the scope of integrated models of care vary across settings, the burden of diseases and health conditions that used to be thought of as ‘Western’ (e.g. cancer, diabetes and heart disease) is increasing rapidly in LMICs creating a double (or triple) burden of non-communicable diseases alongside traditional communicable, infectious diseases. In addition, a rapidly growing challenge for LMIC health care systems are patients with multiple morbidity (e.g. TB and diabetes) while services have typically been organized around a specific infectious disease.

## Does the impact and process of integration differ across settings?

### Impact of integration

There is scant evidence on the outcomes of integrated care in LMICs. In a review published in 2006, Briggs and Garner found only five published studies of adequate quality. They concluded that there was some evidence of an increase in service utilization and better health outcomes associated with a range of integration initiatives (Briggs and Garner 2006). However, the review noted that no conclusions could be drawn on the cost aspects of integration. It also highlighted that the studies only focused on the supply side and did not give consideration to the demand side, with little account taken in these interventions of patients’ views and experiences.

Similarly, a review conducted by the Global Fund (Mangiaterra 2014) showed that the provison of integrated services increased uptake and coverage of several health interventions and had positive effects on some but not all health outcomes. In particular, there were positive effects when screening programmes were provided with routine services since these led to subsequent increases in the uptake of treatment for under-served populations (Stinson et al. 2013; Bindori et al. 2014). However, the provision of a wider platform of health services such as immunization and HIV services showed mixed results in terms of service uptake. The Global Fund review also found some evidence of efficiency gains, in the case where an increase in uptake of Integrated Community Case Management (iCCM) services led to a reduction in unit costs of treatment (iCCM Symposium Final Meeting Report. 2014). This would generally involve investing in activities to create demand for integrated services in the intended population to ensure and sustain high utilization of those integrated services.

In high-income countries, integrated models of care have been promoted as a means to build a more effective and efficient healthcare system that is more patient-centred and thereby better meets the needs of the populations served (Armitage et al. 2009). There is some evidence that integrated care produces better patient experience (Busse and Stahl 2014; Martinez-Gonzalez et al. 2014; Lemmens et al. 2015). However, compared with ‘usual care’ schemes it seldom seems to lead to improved health outcomes (Mason 2013). Evidence of cost effectiveness is generally scarce and contradictory (Armitage et al. 2009; Busse and Stahl 2014; Nolte and Pitchforth 2014). For example, although some reduction in delayed hospital discharge has been identified, no integrated care scheme seems to have demonstrated a sustained reduction in hospital use such as emergency admissions (Mason 2013). It has been argued that this might be due to schemes often focusing on too small proportion of the patient population (patients deemed at high risk of admission) who are very costly per patient, but who are small in number. As a result, even if the care of such people were transformed, this would not improve efficiency system-wide (Stokes et al. 2016). In this respect, such a narrow targeted approach has much in common with the specific programme-based integration of care more commonly found in LMICs.

### Process of integration

If evidence on outcomes and costs of integrated care is mixed or missing, there is plenty of evidence on the processes of integration, in particular, the perceived barriers and facilitators to implementation of integration. The nature of these seems to be remarkably similar and consistent across settings as shown in Table 3, although there are differences in emphasis.

**Table 3.** Comparison of barriers and facilitators to integrated care in HICs and LMICs

Integration	High-income settings	Lower income settings
Examples of enabling strategies	Joint governance arrangements Joint funding arrangements Integrated budgets and funding designed to align providers’ objectives, reduce incentives to cost shifting and encourage efficiency Integrated shared patient records Co-production with patients Multi-disciplinary teams of professionals Generic workers (e.g. Buurtzorg model of nurse-led care) Inter-organizational and inter-personal relationship-building is critical to building integrated services	Leadership (including political will and explicit implementation strategy) and supportive organizational culture Availability and deployment of appropriately trained and incentivised health workers Good staff morale, motivation and support to overcome structural deficiencies Patient-centred delivery taking into account patients’ complex socio-economic and cultural needs Establishment of a workforce trained to provide a wider range of services at community level (e.g. Health Extension Workers); task shifting Integration of prevention and treatment programmes Integrated care to help ‘normalize’ stigmatized conditions (e.g. HIV, TB)
Examples of key challenges faced	Fragmented health care landscape with weak link with prevention Financial barriers between systems thwart efforts to integrate: funding methods are different for health and long-term/social care in many countries (e.g. in England, health is free while social care is means tested) Financial incentives not aligned across types of providers (e.g. acute, primary health care) Competing for resources preventing collaboration (competition rules) Workforce with high degree of professional specialization Lack of IT inter-operability and restrictive information governance rules Lack of ‘hump’ funds to allow providers to transition to different models of care Health care and social care separated by language, conceptions of health, professional cultures and ways of working Primary and community health care sector under-resourced	Siloed funding and reporting, with donors wanting accountable results for their specific programmes Lack of incentives for well-funded programmes to integrate with poorer ones Lack of negotiating power for under-funded programmes Limited capacity, support for and number of staff Poor and fragmented Health Management Information Systems (HMIS) infrastructure Fragmented, poorly coordinated care across agencies/sectors Primary health care is generally under-resourced

Sources: Armitage et al. (2009), Erens et al. (2016), Leggat and Leatt (1997), Mangiaterra (2014), Maruthappu et al. (2015), van der Klauw et al. (2014), Curry and Ham (2010), Ham et al. (2011), Watt et al. (2016), King’s Fund (2014) and Hung et al. (2016).

In HICs, enabling strategies focus on integration of governance, structures and finances, as well as a strong focus on better patient experience. This also means that challenges faced by HICs in realizing integrated care can be significant in LMICs as tackling systemic issues necessitates strong political support and large investments. In LMICs, the core enabling strategies are dominated by improving leadership and motivation of staff at different levels of the system (from political cadres to frontline staff) to enable structural deficits (which are greater than in HICs) to be overcome. Few systemic reforms to support further integration have been implemented in low-income countries beyond pilot schemes, with the exception of Health Extension Workers in Ethiopia (Teklehaimanot and Teklehaimanot 2013).

Overall, the challenges between settings are rather similar, albeit to different degrees, with fragmentation and competition between services for resources exacerbated by specialized (siloed) programmes and workforces common to both sets of countries. Integration across different agencies and sectors seems particularly difficult, notably in LMICs where funding comes from many different sources rather than being the sub-divisions of a single or small number of largely public sources.

## Discussion: can useful lessons be learned across income settings?

We have seen that integrated care has taken somewhat different forms and approaches in high- vs low-/middle-income health care systems. In LMICs, the focus has been more on developing specific clusters of services (e.g. family planning-HIV, IMCI), communicable disease programmes (e.g. HIV), or services for specific patient groups (e.g. pregnant women), while integration in HICs has been more about better management of a broader group of people with multiple morbidities and/or with complex health needs (e.g. with physical and mental health problems) together with a focus on altering the wider system components that support coordination (e.g. information systems, governance, financing). See Table 2.

These approaches have been driven by different emphases in terms of goals and expectations. Of paramount concern for LMICs has been to use integration to improve the uptake of priority services, with a view to gradually achieving universal health care coverage, while concomitantly increasing efficiency (i.e. achieving more with the same money). In HICs, the focus has been more on changing patterns of use (e.g. the shift from hospital in-patient care to primary and community care), with a view to decreasing the overall cost to the system. It has also involved a stronger focus on individual patient experience and an emphasis on improving quality of care. However, in both high- and low-income settings, the implementation of these initiatives tend to uncover unmet need thereby pushing costs upwards even if some savings can be made elsewhere. Overall, there remains a tension between the objectives of improving patient experience (including satisfaction with being able to access a greater range of services) and cost reduction. These appear to conflict, at least in the short term.

The resulting models of care reflect the different ways in which this tension is played out. Integrated models of care in LMICs tend to be more narrowly focused on health services at the point of delivery, while HICs tend to invest in systemic enablers such as governance, financing, planning and information systems while also developing cross-sectoral collaborative models of front-line work such as multi-disciplinary team working, joint contracting, pooled funding and co-production with patients.

A big question for researchers and practitioners is whether there are lessons that can be learned from HIC integration initiatives that might enable greater continuity of patient-centred care in LMICs beyond the specific packages currently targeted for integration. In particular, do HICs have anything to offer in terms of their experience with care planning and service delivery? The lack of patient focus in integrated care schemes in LMICs contrasts with the strong emphasis on improving experience and higher patient engagement in the development of more integrated care pathways in HICs. This includes new ways of measuring users’ experiences of care. It seems likely that LMICs could benefit from giving the patient perspective greater weight when seeking to increase uptake and equity of service delivery, although there would be challenges because the understanding of, and demand for, integrated, better, or even simply different, services is often limited in LMICs (Mak et al. 2013). The recent World Health Assembly’s (2016) call for strengthening integrated people-centred health services provides further impetus to this suggestion (World Health Organization 2015).

A more critical question is perhaps whether or not it is feasible for resource constrained health systems both in low- and high-income settings to move from a narrow focus on particular target services, populations and diseases towards systems that seek to integrate functions more widely at every level. Currently, this is proving particularly challenging as so many health care programmes in LMICs remain vertically funded and managed. As a result, the supporting systems such as information, finance, contracting and training also tend to be separate, often spurred by external funding agencies. With the burden of disease becoming more complex and co-morbidities increasing rapidly, LMICs need to focus more on integrating systems as well as services. The experience of HICs could be very valuable in this regard.

In turn, it is possible that HICs can also learn from LMIC experiences, in particular, of improving communication and coordination between sectors and agencies even when formal bridging structures do not exist. For example, as Mayhew et al illustrate in this Supplement (Mayhew et al. 2017), front line staff in LMICs with motivation and support (from peers and managers) are able to take the initiative to make connections without having to wait for wider system change. Another area of learning for HICs which tend to have an extremely specialized health care workforce is to explore how LMICs have been able to extend specialized health care workers’ skills to provide more holistic services through inter-professional education and collaborative practice, including, in some cases, by formally task-shifting. Finally, population-wide prevention and health promotion interventions are often directly integrated with treatment programmes in LMICs (e.g. screening, provision of nutritional supplements and de-worming treatment) when contrasted with separate prevention, health promotion and individual treatment programmes that predominate in HICs.

## Conclusions

This commentary has made clearer how integrated care models differ across HICs and LMICs in scope, models of care and expectations.

There is still insufficient convincing evidence that integration has a significant impact on improving health outcomes or cost effectiveness in any setting. This is partly due to the very wide range and heterogeneity of integrated schemes, their complexity and the difficulties of rigorously evaluating these schemes.

However, there are potential lessons from one type of setting to another that are worth exploring further. This commentary seeks to spark wider debate about what lessons could—and should be drawn between income settings while being cognisant of—but not constrained by—the contextual differences. Through pooling and discussing insights across settings, we can maximize our understanding of how and why health systems and their staff are better able to provide holistic patient-centred care ultimately leading to better health outcomes. This is consistent with the recent WHO (2015) strategy to promote people-centred integrated care. However, introducing and scaling up integrated care will need to be undertaken carefully through piloting and evaluation of potentially promising integrated care schemes, especially in highly resource-constrained settings. Evidence of the benefits of various forms of integrated care remains insufficient to support rapid scale-up. Among key research questions that need to be addressed by countries and international organizations alike are: how patient-centred are integrated care schemes; what is their cost-effectiveness over the medium term; and what are their effects on the wider local health system? These are challenging but important questions if the global community wants to continue supporting more integration of care. This will need commitment both politically and financially. Finally, it will be critical for researchers from currently very separate parts of health services and systems research to start a dialogue on how to share methods and substantive knowledge to evaluate integrated care comparatively in a wider range of settings, and thus provide better evidence to policy-makers.

Box What high- and low-income countries can learn from each other

Lower income countries: what they can learn

- More patient focus; co-production of services
- Measuring patient experience
- More emphasis on, and evaluation of, quality of care
- Re-designing systems rather than specific services
- Promoting coordination between sectors and funding agencies
- Prioritizing funding to primary care and community care
- Prioritizing provider payment mechanisms that promote integrated care and reduce cost shifting (e.g. weighted capitation funding)

High-income countries: what they can learn

- Decrease professional barriers and promote joint working/task broadening/use of generic workers
- Emphasis on integration of health promotion with prevention and treatment services

*Conflict of interest statement.* None declared.
